# Caspofungin versus anidulafungin in patients with invasive candidiasis: a retrospective study with propensity-score-matched analysis

**DOI:** 10.1177/20499361251344777

**Published:** 2025-08-02

**Authors:** Reem Hasan Elajez, Dana Bakdach, Sara Al Balushi, Ahmed Zaqout, Rand Alattar, Tasneem Abdallah, Waleed Awouda, Godwin Wilson, Walid Al-Wali, Emad Ibrahim, Hussam Alsoub

**Affiliations:** Pharmacy Department, Hamad General Hospital, Hamad Medical Corporation, P.O. Box 3050, Doha, Qatar; Pharmacy Department, Hamad General Hospital, Hamad Medical Corporation, Doha, Qatar; Epidemiology Section, Communicable Disease Centre, Hamad Medical Corporation, Doha, Qatar; Division of Infectious Diseases, Department of Medicine, Communicable Disease Centre, Hamad Medical Corporation, Doha, Qatar; Pharmacy Department, Communicable Disease Center, Hamad Medical Corporation, Doha, Qatar; Division of Infectious Diseases, Department of Medicine, Communicable Disease Centre, Hamad Medical Corporation, Doha, Qatar; Division of Infectious Diseases, Department of Medicine, Communicable Disease Centre, Hamad Medical Corporation, Doha, Qatar; Department of Laboratory Medicine and Pathology, Hamad Medical Corporation, Doha, Qatar; Department of Laboratory Medicine and Pathology, Hamad Medical Corporation, Doha, Qatar; Department of Laboratory Medicine and Pathology, Hamad Medical Corporation, Doha, Qatar; Biomedical Research Center, Qatar University, Doha, Qatar; Division of Infectious Diseases, Department of Medicine, Communicable Disease Centre, Hamad Medical Corporation, Doha, Qatar

**Keywords:** anidulafungin, caspofungin, invasive candidiasis

## Abstract

**Background:**

Echinocandins are recommended as an initial treatment for invasive candidiasis. Although safety and efficacy profiles of both anidulafungin and caspofungin are well established, direct head-to-head comparisons have not been reported before.

**Objective:**

Compare efficacy and safety of anidulafungin versus caspofungin among patients with invasive candidiasis.

**Design:**

Retrospective observational study.

**Methods:**

Adult patients with invasive candidiasis who were treated with either anidulafungin or caspofungin for ⩾5 days were retrospectively reviewed over a period of 6 years. The primary endpoint was global response, defined as clinical and microbiological success at the end of treatment duration.

**Results:**

A total of 223 patients who received either anidulafungin (*n* = 176) or caspofungin (*n* = 47) were initially included. Propensity score matching (based on age, malignancy, level of care, presence of candidemia, and other factors) was performed to improve comparability of the two groups. As a result, 32 patients in the caspofungin arm and 79 patients in the anidulafungin arm were included in the final analysis. Around three-quarters of the cohort had candidemia, and the most common isolated *Candida* species were *C. albicans* and *C. glabrata*. Response rates were comparable between both groups, with the primary outcome of global response showing no significant difference (56.3% for the caspofungin group vs 63.3% for anidulafungin, *p* = 0.490). Similarly, no differences between the two groups were observed in terms of 90-day all-cause mortality (*p* = 0.672) or any other secondary endpoints.

**Conclusion:**

Our data suggest that anidulafungin and caspofungin have comparable global response among patients with invasive candidiasis. Additionally, both studied echinocandins showed no significant difference in 90-day all-cause mortality. However, due to the limited sample size, larger studies are needed to confirm these results.

## Background

Over the last few decades, invasive candidiasis (IC) has been recognized as one of the leading causes of morbidity and mortality within the healthcare system particularly among the critically ill population.^1,2^ While commonly linked to candidemia, IC in fact involves both candidemia and other forms of deep-seated candidiasis, in which *Candida* isolates are obtained from sterile tissue samples.^3^

Echinocandins have been proven to exert activity against a broad spectrum of *Candida* species including *C. glabrata* and *C. krusei*.^4^ As a class, echinocandins include Caspofungin, Anidulafungin, and Micafungin, with a fourth agent, Rezafungin, being recently approved by US Food and Drug Administration (FDA) in March 2023.^5^ The differences between these drugs are primarily related to their FDA-approved indications, pharmacokinetic profiles, and safety parameters.^6,7^ Different guidelines including Infectious Diseases Society of America and European Society of Clinical Microbiology and Infectious Diseases recommend echinocandins as the preferred initial treatment class for IC,^8–10^ yet no specifications are made as for one echinocandin over the other. Given its non-hepatic degradation, some clinicians tend to prefer anidulafungin over other echinocandins, with the perception of being less likely to cause hepatotoxicity or worsening any pre-existing liver damage.^11,12^ Furthermore, in a pharmacoeconomic evaluation conducted by Auzinger et al.,^13^ anidulafungin was found to be associated with cost-saving and lower total costs compared to caspofungin or micafungin. Most of the available literature to-date compared micafungin to other echinocandins (caspofungin or anidulafungin) in terms of efficacy and/or safety, with most of the data being based non-ICU population, focusing on safety outcomes, or rarely reporting clinically relevant outcomes (e.g., mortality).^14,15^ However, head-to-head efficacy and/or safety comparisons between anidulafungin and caspofungin when used for the management of IC are still lacking despite its proven clinical efficacy. Therefore, the aim of this study was to compare the clinical efficacy and safety outcomes of anidulafungin versus caspofungin among patients treated for IC.

## Methods

### Study design and setting

This was a retrospective observational study, with propensity-score matched analysis. Utilized data were retrieved from the medical records of patients treated with either caspofungin or anidulafungin over a period of 6 years (2016–2021), in the eight main governmental hospitals in Qatar (Hamad General Hospital (HGH), Al Wakra Hospital (AWH), Al Khor Hospital (AKH), Rumaila Hospital (RH), Heart Hospital (HH), Qatar Rehabilitation Institute (QRI), Women’s Wellness and Research Center (WWRC), and Hazm Mebaireek General Hospital (HMGH)).

### Patients/medications

All adult patients diagnosed with IC and had received either caspofungin or anidulafungin for a duration of at least 5 days were considered for participation. The decision to start an echinocandin was made by the attending physician in collaboration with the infectious disease team. The administered doses of caspofungin or anidulafungin were in adherence with the recommended dosage regimens tailored to the patient’s specific conditions (e.g., indications, liver disease, and weight) ranging from 35 to 70 mg daily for caspofungin and 100 to 200 mg for anidulafungin. Patients were included at their first episode of IC. IC in this study was defined as *Candida* infections that include either: (1) Candidemia: at least one positive blood culture indicating the presence of any *Candida* spp., or (2) Candidiasis: a positive culture of *Candida* spp. obtained from a normally sterile site (e.g., pleural, peritoneal fluid), accompanied by one or more of the following signs and symptoms of infection: fever/hypothermia, hypotension, localized signs and symptoms of inflammation, or radiological findings (collection or abscess, such as in intra-abdominal infections or empyema) suggestive of IC.^13,14^ Patients who had received any systemic antifungal medication for more than 48 h within the preceding 14 days were excluded from the study.

### Outcomes

The primary endpoint was global response, which encompassed both clinical and mycological success at the end of echinocandin treatment. Clinical success was defined as the resolution of all signs, symptoms, and abnormal radiographic findings (i.e., collection size) associated with *Candida* spp. Infection. For patients with candidemia, mycological success was defined as confirmed eradication with at least one repeated blood culture with negative results. Blood cultures are typically repeated every 48 h until eradication is documented, guiding treatment duration based on concurrent infections or complications (with a minimum of 14 days from the first negative culture). For patients with IC other than candidemia, mycological success was defined as either (a) confirmed eradication: patient with a follow-up culture or biopsy confirming eradication, or (b) presumed eradication: patient had clinical response but had no follow-up culture or biopsy done to confirm the eradication. Treatment failure was defined as either presence of *Candida* spp. on repeated cultures among patients who had received studied echinocandins or no noticeable clinical improvement in the patient’s condition related to candida infection, regardless of the culture findings.

Secondary endpoints included: all-cause mortality at both 30 and 90 days after the start of echinocandin therapy. Safety analysis was assessed based on incidence of liver toxicity (defined as an elevation of Alanine aminotransferase and/or aspartate aminotransferase levels exceeding three times the upper normal limit in cases where the baseline levels were initially normal; and more than 1.5 times baseline reading if baseline level was abnormal, aligning with the adverse events criteria outlined in the Common Terminology Criteria for Adverse Events, Version 5.^16^

### Statistical analysis

Propensity score matching was used, in which matching data included age, malignancy, level of care ((Intensive Care Unit) vs non-ICU), Sequential Organ Failure Assessment (SOFA) score, Charlson Comorbidity Index, administration of immunosuppressant or broad-spectrum antibiotics, presence of candidemia, and source control. Patients were matched in a 1:3 ratio (caspofungin:anidulafungin) using the nearest neighbor matching method, with caliper of 0.2.

Categorical variables were presented as counts and percentages, while normally distributed continuous variables were summarized as means and standard deviations. Non-normally distributed variables were presented using the median, along with the 25th and 75th percentiles. The Shapiro–Wilk test along with histograms was used to test data for normality. *T*-test was employed for normally distributed variables, whereas the Mann–Whitney test was utilized for non-parametric variables. Categorical variables were analyzed using Chi-square test. Survival analysis was conducted using Kaplan–Meier survival curves, illustrating 30- and 90-day mortality rates. The log-rank test was applied to evaluate the survival data. Univariable and multivariable analyses were conducted to evaluate the impact of covariates on the outcomes of interest. A multivariable logistic regression model was constructed to identify the factors associated with the outcomes using stepwise regression. First, a univariate analysis was conducted to include variables with *p*-values < 0.25 in the initial model; then, after adjusting for all variables, variables with *p*-values ⩽ 0.05 were kept in the final model along with the clinically relevant variables even if not statically significant. Model performance was assessed using operating characteristics curves. Statistical significance was defined as a two-tailed *p*-value of less than 0.05. All data analysis was performed using Stata/SE 14.2.

## Results

### Baseline characteristics

A total of 223 patients with IC who had received either caspofungin (*n* = 47) or anidulafungin (*n* = 176) were initially included. Baseline demographics were comparable between the two treatment arms except for the incidence of diabetes mellitus and the duration of hospitalization, which was notably higher among the anidulafungin group (Supplemental Table 1).

Due to imbalanced sample size between both groups, propensity score matching was employed (with ratio of 1:3), resulting in 32 patients on caspofungin and 79 on anidulafungin who were finally analyzed. After matching, demographics and clinical characteristics between both groups were more balanced (Table 1). In addition to the factors used for matching, similarities were also observed in demographics (weight, gender), comorbidities, and infection/treatment-related characteristics (*p* > 0.05 for all comparisons). Candidemia was observed in around three-quarters of the patients at baseline, while intra-abdominal infection was the most common non-candidemia cause of IC. The most isolated *Candida* species were *C. albicans* and *C. glabrata*, together accounting for over 50% of all isolates. The median duration of echinocandins treatment was 14 days for both arms. Source control (i.e., either line removal in case of line-related infection, or drainage of the collection) was achieved more often in the anidulafungin arm (88.6%) compared to the caspofungin arm (78.1%), although this difference was not statistically significant.

**Table 1.** Baseline demographic and clinical characteristics (propensity score matched cohort).

Demographic and clinical characteristics	Caspofungin *N* = 32 (29%)	Anidulafungin *N* = 79 (71%)	*p* Value
Baseline patient characteristics^a^			
Age (years), mean ± SD	54 ± 18	53 ± 17	0.73
Weight (kg), median (IQR)	71 (62–81)	75 (64–86)	0.47
Malignancy (solid/hematology)	7 (21.9%)	14 (17.7%)	0.61
Gender (Male)	24 (75%)	61 (77.2%)	0.80
Liver failure	2 (6.3%)	7 (8.9%)	1
Neutropenia^b^	1 (3.1%)	4 (5%)	1
Recent surgery^c^	13 (40.6%)	25 (31.6%)	0.37
Mechanical ventilation	16 (50%)	49 (62%)	0.24
Diabetes mellitus	8 (25%)	14 (17.7%)	0.38
Immunosuppressant^d^	2 (6.3%)	4(5%)	1
On broad spectrum antibiotics	27 (84.4%)	73 (92.4%)	0.20
Renal replacement therapy	12 (37.5%)	23 (29.1%)	0.39
Total parenteral nutrition (TPN)	10 (31.3%)	20(25.3%)	0.52
Central venous catheters	26 (81.3%)	61 (77.2%)	0.64
Charlson Comorbidity Index, median (IQR)	4 (2–8)	3 (0–8)	0.29
Length of hospital stay (days), median (IQR)	8 (2–27)	15 (3–35)	0.12
ICU admission	18 (56.3%)	48 (60.8%)	0.66
Admission SOFA score, median (IQR)	8 (1–16)	9 (1–18)	0.43
Infection/treatment Characteristics			
Indication/site of infection^e^			
Candidemia	25 (78.1%)	60 (75.9%)	0.81
Intra-abdominal infection	12 (37.5%)	23(29.1%)	0.39
Pleural effusion/empyema	8 (25%)	11 (13.9%)	0.16
*Candida* species			0.84
*Candida glabrata*	8 (25%)	15 (19%)	
*Candida albicans*	9 (28.1%)	26 (32.9%)	
*Candida auris*	2 (6.3%)	6 (7.6%)	
*Candida tropicalis*	3 (9.3%)	9 (11.4%)	
*Candida parapsilosis*	5 (15.6%)	9 (11.4%)	
*Candida krusei*	2 (6.3%)	2 (2.5%)	
Other *candida* species	1 (3.1%)	7(8.9%)	
Multiple *candida* species	2 (6.3%)	5 (6.3%)	
Repeated culture			0.16
Cleared/Negative	18 (56.3%)	58 (73.4%)	
Persistently positive	4 (12.5%)	8 (10.1%)	
Not done	10 (31.2%)	13 (16.5%)	
Reason of early discontinuation of antifungal^e^			
Failure	1 (3.1%)	8 (10.1%)	0.44
Side effect	0 (0%)	2 (2.5%)	0.36
Death/ discharge	9 (28.1%)	19 (24%)	0.65
Antifungal treatment duration (days), median (IQR)	14 (8–17)	14 (10–17)	0.876

a Data presented as count (%) unless indicated otherwise.

b Neutropenia: an ANC of less than 500 cells/µL.

c Recent surgery: any surgical procedure performed within 30 days.

d Immunosuppressant: administration of corticosteroids (equivalent to ⩾20 mg/day of prednisone for ⩾2 weeks), chemotherapy, or other immunosuppressive agents within the past 30 days.

e Multiple indications/reasons, the sum may not add up to 100%.

ANC, absolute neutrophil count; ICU, intensive care unit; IQR, interquartile range; SD, standard deviation.

### Primary outcome

Of all reviewed patients before matching (*n* = 223), the primary outcome of global response was achieved by 26 (55.3%) of the 47 patients in the caspofungin arm, and 115 (65.3%) of the 176 patients in the anidulafungin arm (*p*-value 0.206; Table 2). Similar results were observed after matching, with global response being observed in 18 patients (56.3%) in the caspofungin group, compared to 50 patients (63.3%) in the anidulafungin group (*p*-value 0.490; Table 2). Upon examining clinical and mycological success independently, anidulafungin was observed to have higher success rates compared to the caspofungin group, yet not reaching statistical significance (Table 2).

**Table 2.** Primary and secondary outcomes before and after propensity score matching.

Primary and secondary outcomes	Nonmatched sample			Matched sample		
	Caspofungin (*n* = 47)	Anidulafungin (*n* = 176)	*p*-Value	Caspofungin (*n* = 32)	Anidulafungin (*n* = 79)	*p*-Value
Global response	26 (55.3%)	115 (65.3%)	0.206	18 (56.3%)	50 (63.3%)	0.490
Clinical success	27 (57.4%)	118 (67%)	0.220	18 (56.3%)	52 (65.8%)	0.344
Mycological success	27 (57.4%)	124 (70.5%)	0.208	19 (59.4%)	59 (74.7%)	0.411
All-cause mortality						
30-day mortality	14 (29.8%)	51 (29%)	0.914	9 (28.1%)	24 (30.4%)	0.814
90-day mortality	23 (48.9%)	83 (47.2%)	0.828	16 (50%)	36 (45.6%)	0.672
Liver enzyme elevation	9 (19.1%)	37 (21%)	0.477	9 (28.1%)	30 (38%)	0.192

### Secondary outcomes

**Figure 1.**
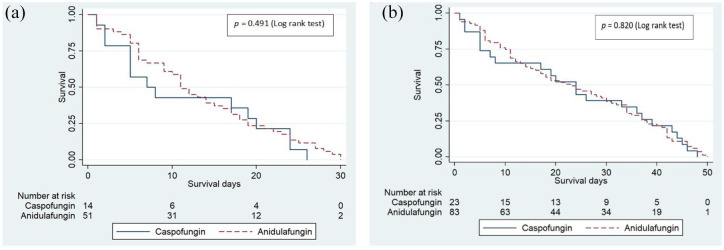
Kaplan–Meier survival curve up to (a) 30 days (up) and (b) 90 days (down) after initiation of an echinocandin.

All-cause mortality was comparable between both groups at 30 and 90 days. No significant disparities in all-cause mortality between the two groups were noted after matching. For instance, 30-day mortality rate was 28.1% for the caspofungin group and 30.4% for the anidulafungin group (*p* = 0.814), while 90-day mortality rate was 50% and 45.6%, respectively (*p* = 0.672; Table 2). The log-rank test indicated that there was no significant difference in survival between caspofungin and anidulafungin groups at both 30 days (*p* = 0.491) and 90 days (*p* = 0.820) (Figure 1(a) and (b)).

In terms of safety, only two patients in the anidulafungin arm experienced side effects necessitating therapy discontinuation, whereas none were reported in the caspofungin arm. The two cases reported with anidulafungin were: generalized maculopapular skin rash, identified as “toxic pustuloderma” in one patient, and persistent hypokalemia not responding to treatment. After matching, liver toxicity differed notably, occurring in nine patients (28.1%) in the caspofungin group and 30 patients (38%) in the anidulafungin group. However, again, such difference was not statistically significant (*p* = 0.192) (Table 2).

The results of multivariable logistic regression analyses for primary and secondary outcomes in the propensity score-matched cohort, as well as for the global response in the same cohort, are presented in Supplemental Tables 2 and 3, respectively. The odds ratios and corresponding 95% confidence intervals showed no significant differences in global response, clinical success, mycological success, 30-day mortality, and 90-day mortality between the two treatment groups.

## Discussion

To our knowledge, this is the first published head-to-head comparison between anidulafungin and caspofungin when utilized for the treatment of IC. The results of this analysis suggest similar efficacy and safety outcomes associated with the use of either anidulafungin or caspofungin.

Among patients with candidemia, antifungals are usually recommended for a total duration of 2 weeks after the documented clearance of *Candida* species from bloodstream provided that no other indications exist for longer duration (e.g., endocarditis, osteomyelitis, etc.).^8^ Our observed median duration of 14 days of echinocandins use in this cohort is going in-line with the aforementioned recommendation, given that candidemia accounted for more than 75% of IC cases included.

The findings of this study align with the robust efficacy and safety profiles of caspofungin and anidulafungin reported among other trials.^12–15,17,18^ Yet, the overall response rates in this study are slightly different than those reported in previous studies, with the difference being more pronounced in the caspofungin group compared to the anidulafungin group. For instance, in 2009, Mills et al.^12^ reported an overall success rate of 76.1% associated with caspofungin use for the management of IC in their meta-analysis. Similarly, when caspofungin was compared to micafungin, Pappas et al.^15^ reported a success rate of 72.3% in the caspofungin arm. The observed lower success rate seen in this current study (i.e., 56.3%) might have been driven by different factors, including smaller sample size of the caspofungin arm, low rates of appropriate source control, and/or relatively high percentage of persistently positive cultures. Nonetheless, despite such numerically observed lower success rates seen in the caspofungin arm, it did not translate into a significant difference in either primary or secondary endpoints when compared to anidulafungin.

In terms of safety, transient elevation of liver enzymes during echinocandin therapy has been reported to occur in 2%–15% of patients previously.^19^ In this study, we have observed higher rates of liver enzymes’ elevations across both arms reaching up to 38%. This difference might have been explained by the fact that more than half of the included cohort were critically ill admitted to the ICU; hence, other factors (e.g., concomitant therapies, hypotension, or sepsis) could have contributed to the higher observed incidence, especially that liver enzymes elevations were observed in the overall cohort rather than being attributed to the specific echinocandin used.^20^ Similar rates of elevated liver enzymes were described by van der Geest et al.,^14^ when echinocandins were utilized for IC management among critically ill population.

This study has some limitations. First, within our cohort, the assessment of acquired echinocandin resistance was not conducted among subsequent cultures during follow-up. Recent studies have shown increased rates of acquired echinocandin resistance during therapy in initially susceptible *C. glabrata* species.^21–23^ While *C. glabrata* was the second most isolated species, data about acquired resistance were not collected, and hence, effects on treatment failure or persistence positivity could not be evaluated. Likewise, information on infection relapse or recurrence was also not collected. Second, we were unable to conduct a refined analysis of echinocandin-induced hepatotoxicity considering different contributing clinical factors that could have potentially led to the observed increased occurrence of liver enzymes’ derangements in our study. Finally, and most notably, having a relatively modest sample size coupled with the retrospective nature of the study could have impacted our results. Due to the retrospective nature of the study, the time from symptom onset to treatment initiation could not be assessed, representing a limitation of the study. However, with regard to the time interval between culture collection and initiation of antifungal therapy, no statistically significant difference was observed between the groups. This is likely attributable to the high prevalence of candidemia among the study population (75%), where antifungal treatment was promptly initiated upon detection of Candida in preliminary stain results, even prior to definitive species identification. Consequently, differences in this interval between the groups were minimal. For the sample size, while the initial study plan was aiming for a 3-year period of retrospective review, the caspofungin arm was found to be very small. With the approval of the medical research center, we extended the duration of the retrieved cases to 6 years and include all governmental hospitals within the country. Nevertheless, the caspofungin sample size remained small, thus reflecting lower rates of prescribers’ utilization caspofungin among proven cases on IC leading to the study being underpowered. Nevertheless, this does not necessarily indicate the absence of the clinical importance of the results; rather, it underscores the preliminary nature of the findings and suggests that it may serve as hypothesis-generating, with potential variability depending on the formulary echinocandin and local ecological factors. The observed outcomes may be deemed preliminary, emphasizing the need for upcoming larger studies to offer more robust evidence regarding the effects observed.

## Conclusion

Considering the comparable clinical and mycological response rate, coupled with similar rates of all-cause mortality and safety profile, our findings suggest that anidulafungin and caspofungin may be regarded as similarly effective for the treatment of IC. Nonetheless, given the small sample size of the current study, further prospective larger studies are still warranted to confirm such observations.

## Supplemental Material

sj-docx-1-tai-10.1177_20499361251344777 – Supplemental material for Caspofungin versus anidulafungin in patients with invasive candidiasis: a retrospective study with propensity-score-matched analysis Supplemental material, sj-docx-1-tai-10.1177_20499361251344777 for Caspofungin versus anidulafungin in patients with invasive candidiasis: a retrospective study with propensity-score-matched analysis by Reem Hasan Elajez, Dana Bakdach, Sara Al Balushi, Ahmed Zaqout, Rand Alattar, Tasneem Abdallah, Waleed Awouda, Godwin Wilson, Walid Al-Wali, Emad Ibrahim and Hussam Alsoub in Therapeutic Advances in Infectious Disease

## Declarations

**Ethics approval and consent to participate:** The study was approved and conducted with adherence to the Institutional Review Board/Human Subjects Research Committee’s requirements at the Medical Research Center, Hamad Medical Corporation, Qatar (MRC-01-19-331 & MRC-01-21-992). HMC-IRB approval covers all sites included in the study. IRB granted a waiver of consent for this minimal-risk retrospective study using de-identified patient data, with no direct patient interaction, in compliance with ethical guidelines.

**Consent for publication:** Not applicable.

**Availability of data and materials:** Not applicable.
